# The Impact of Birth Year on the Coming Out Process for Transgender and Gender Diverse Individuals from Austria

**DOI:** 10.1007/s10508-025-03397-8

**Published:** 2026-03-02

**Authors:** Katharina Feil, Thessa Seeland, Sarah Maier, David Riedl, Anna Lena Zippl, Bettina Toth

**Affiliations:** 1 Department of Gynecological Endocrinology and Reproductive Medicine, Medical University of Innsbruck, 6020 Innsbruck, Austria; 2 Institute of Clinical Epidemiology, Public Health, Health Economics, Medical Statistics and Informatics, Medical University of Innsbruck, Innsbruck, Austria; 3 Department of Psychiatry, Psychotherapy, Psychosomatics and Medical Psychology, University Hospital of Psychiatry II, Medical University of Innsbruck, Innsbruck, Austria

**Keywords:** Gender minority, Gender identity, Milestones, Hormone therapy, Transgender, Gender dysphoria

## Abstract

This study examined age-related cohort differences in the timing of three key gender identity milestones (first desire to transition, coming out, and initiation of hormone therapy) across gender identities. Data from 212 adult transgender and gender diverse (TGD) individuals (54.2% trans male, 42.5% trans female, 3.3% gender diverse; age 20–76 years) were collected at a single center between 2017 and 2021. Gender diverse participants, defined as those with identities outside the binary, were grouped accordingly. Selected items relating to gender identity milestones and sociodemographic information were drawn from the Innsbruck Quality of Life Questionnaire. Linear regression revealed significant negative correlations between year of birth and age at first desire to transition (*B* = − 0.57, *p* < 0.001), coming out (*B* = − 0.77, *p* < 0.001), and hormone therapy initiation (*B* = − 0.89, *p* < 0.001), indicating earlier milestone attainment in later birth cohorts. Logistic regression showed that earlier birth year significantly predicted higher odds of identifying as a trans woman (OR = 0.95, *p* < 0.001). Analysis of birth year distribution by gender identity further indicated an increasing trend in individuals seeking hormone therapy, with younger birth years more prevalent among trans men. An association was demonstrated between year of birth and time spans between gender identity milestones, showing a shortening in time intervals in younger participants. Educational attainment in the study cohort was lower than in the general Austrian population. These findings suggest a temporal shift toward earlier realization and affirmation of gender identity among TGD individuals.

## Introduction

The process of coming out or openly acknowledging one’s transgender or gender diverse (TGD) identity is a critical and multifaceted experience (Brumbaugh-Johnson & Hull, 2019; Puckett et al., 2022; Scandurra et al., 2021; Taube & Mussap, 2024). It ranges from recognizing and accepting one’s own TGD identity, disclosing it to the social environment and the public, to initiating transition toward one’s affirmed gender. In this context “coming out” refers to the act of sharing one’s TGD identity with others, while “transition” may include social transition (e.g., changing name and/or legal status, changing appearance) and medical transition (e.g., gender-affirming hormone therapy (GAHT), hair removal, speech therapy, gender-affirming surgery) depending on individual goals and needs. While these processes may overlap, it is important to note that coming out and transitioning are not synonymous. Coming out may precede, accompany, or follow various aspects of social or medical transition. It is imperative to comprehend the complex interplay between the process of coming out and transition, particularly in the context of the persistent influence of minority stressors. The stressors encompass discrimination, the prospect of rejection, and the pressure to conform to binary gender norms (Hendricks & Testa, 2012; Meyer, 2003). These factors may also interact with age, as generational differences in exposure to stigma and acceptance could shape when and how TGD individuals feel safe to come out or transition.

The fear of stigma or violence often requires TGD individuals to make ongoing, complex decisions regarding disclosure and self-presentation (Brumbaugh-Johnson & Hull, 2019; Enogieru et al., 2024). Non-binary TGD individuals are particularly vulnerable to discrimination and minority stress in a binary environment (Colson et al., 2024; Enogieru et al., 2024). Within this conceptual framework, it is reasonable to hypothesize that both personal and contextual factors determine how, when, and to whom TGD people choose to disclose their identity or pursue transition steps. One of the significant factors under consideration is age.

Maguen et al. (2007) examined the prevalence and predictors of coming out in TGD individuals, finding that younger age is associated with higher rates of disclosure. The authors attributed this to evolving societal attitudes and greater community-level support. Older TGD individuals may have grown up in eras characterized by greater stigma and less acceptance of gender diversity, which may have affected their willingness and ability to come out. Conversely, younger TGD individuals benefit from recent advances in LGBTQ+ rights and representation, although they still face significant challenges and discrimination. This generational context underscores the importance of considering age as a critical factor in the coming out process. In addition, understanding how age affects this process is important for developing supportive environments and interventions that meet the diverse needs of TGD individuals at different stages of life.

Only few studies have examined milestones in gender identity development of TGD individuals in relation to age (Puckett et al., 2022; Scandurra et al., 2021; Wilkinson et al., 2018). A US-based cohort of 3,796 TGD individuals was examined to determine the impact of generational categories on milestones and their influence on educational attainment. The study used data from the National Transgender Discrimination Survey collected between September 2008 and March 2009 and recorded the recognition of gender difference, the first recognition of TGD identity, and the age at which individuals first lived as TGD. The study revealed that individuals who first encountered transgender identity milestones during adolescence exhibited a lower level of educational attainment compared to those who first encountered such milestones at other life stages (Wilkinson et al., 2018).The impact of first realization of a TGD identity, self-identification as TGD, and the act of coming out on mental health was explored in an Italian cohort of 197 TGD individuals. Younger participants were more likely to identify as trans men and to disclose their identity earlier than older cohorts but reported higher negative expectations and lower levels of disclosure. Finally, younger cohorts had poorer mental health (Scandurra et al., 2021). Another US-based study examined the ages at which various milestones were reached (first experiencing gender incongruence, living as one’s affirmed gender, and accessing gender-affirming medical care), broken down by gender and generation. The study also examined how these milestones relate to stress and mental health. The online study surveyed 695 transgender individuals. Older generations were more likely to identify as trans women than younger generations, who displayed a greater diversity of identities. Trans women reported transitioning at a later age than other gender groups. Older generations reported later ages for milestones compared to younger generations. Younger generations reported higher levels of internalized stigma, anxiety, and depression than older generations (Puckett et al., 2022).

However, research on the temporal aspects of coming out in TGD individuals remains limited, particularly with regard to the time interval between different steps in the coming out process and the transition. Existing studies have examined generational differences in the timing of individual milestones, but none has modeled both birth year and gender identity as predictors of the time intervals between milestones. Therefore, the aim of this study was to investigate the chronological process of coming out in TGD individuals, focusing on the first desire to transition, the act of coming out, and the onset of GAHT across different birth years and gender identities.

Understanding how the timing and sequence of coming out milestones differ across age groups is not only theoretically relevant for models of gender identity development but also has practical implications for clinical and community support. Age-related differences may influence access to care, readiness for transition, and exposure to minority stress. Additionally, various sociocultural influences likely contribute to cohort effects and the timing of gender identity milestones. These influences include evolving gender identity norms, changes in legal and diagnostic frameworks, increased visibility through online communities, and adaptations in medical pathways. Clarifying these dynamics can therefore inform age-sensitive interventions and improve psychosocial support for TGD individuals across the lifespan.

This study addressed this gap by investigating age-related differences in the timing and interval between key milestones (first desire to transition, coming out, and initiation of gender-affirming hormone therapy) across gender identities in an adult TGD cohort. By examining the chronological process and potential cohort effects, our aim was to provide a more nuanced understanding of identity development in TGD individuals.

## Method

### Participants

Participants for this study were recruited during consultation at a center specializing in endocrinological TGD care over a period of 4 years or online.

Inclusion criteria ensured that participants were:

- 18 years of age or older
- Identified as transgender or gender diverse (outside the gender binary, e.g., non-binary, agender, genderqueer, gender nonconforming or gender fluid)
- Undergoing or planning to undergo GAHT
- Able to provide informed consent

Those excluded from the study were:

- Minors
- Individuals with contraindications to GAHT

### Measures and Procedure

Between 2017 and 2021, data were collected at a single center specializing in endocrinological care for TGD adolescents and adults in Innsbruck/Austria. For this study, selected items relating to gender identity milestones and sociodemographic characteristics were drawn from the Innsbruck Transgender Quality of Life Questionnaire (iTransQoL), a validated instrument for assessing quality of life in TGD individuals during GAHT (Feil et al., 2022). The collected information included age, gender identity, sexual orientation in affirmed gender, nationality, and education level. Three key milestones in TGD identity development were assessed by asking participants to indicate:

1. The year of first desire to transition (“Since when have you felt the wish to transition?”).
2. The year of coming out (“When did you start coming out?”).
3. The year in which gender-affirming hormone therapy was initiated (“Since when have you been on hormone therapy?”).

The full questionnaire consists of 38 items that cover various aspects of quality of life specific to TGD individuals, including personal and emotional well-being, social and occupational support, body image and self-confidence. Study participants completed the questionnaire either during consultation visits at the center or via online survey platform.

### Statistical Analysis

A descriptive demographic analysis of the study population was conducted. Age calculations used a standardized reference date of June 5, 2024, to ensure consistency across participants. This approach was necessary because not all previous data included questionnaire completion dates.

Mean age and standard deviation (SD) at which the participants experienced the three key gender identity development (GID) milestones (desire to transition, coming out, and initiation of gender-affirming hormone therapy) were calculated separately for trans men, trans women, and gender diverse. Ages at GID milestones of trans men and trans women were compared using Mann–Whitney-U tests.

Time intervals between GID milestones were calculated by subtracting each participant’s respective milestone years. Means (SD) were calculated for three time periods: (1) time between desire to transition and coming out, (2) coming out and initiation of hormone therapy, and (3) desire to transition and initiation of hormone therapy.

Univariable linear regression was used to analyze the associations of the year of birth with the metric outcome variables (age at GID milestones, time intervals between GID milestones). Binary logistic regression was used to assess the association of the year of birth with gender identities (trans woman vs. trans men and gender diverse individuals). Correlation coefficients and odds ratios, respectively, with 95% confidence intervals (CI) and *p*-values were reported. For graphical illustrations, boxplots, scatter plots, and histograms were used. Patterns of missing data were observed, primarily in responses to questions about GID milestones, see Appendix A. All statistical analyses were performed using IMB SPSS Statistics 28.0.0.0. (IBM Corp., Armonk, NY) with significance set at alpha = 0.05.

## Results

The study sample consisted of 212 TGD participants. The gender identity distribution revealed that 54.2% (*n* = 115) identified as trans men, 42.5% (*n* = 90) as trans women, and 3.3% (*n* = 7) as gender diverse (*n* = 3 assigned female at birth, *n* = 4 assigned male at birth). Participants ranged in age from 20 to 76 (min–max) years, with a mean of 31.3 years (10.4 SD). Regarding educational attainment, the most common qualification was an apprenticeship (32.9%), followed by a high school diploma (26.2%). Table 1 provides a comprehensive summary of the demographic profile of the study population.

**Table 1:** Sample demographics

Characteristics	Trans men (*n* = 115)	Trans women (*n* = 90)	Gender diverse (*n* = 7)
Age, Median (range)	26 (20–75)	30 (20–76)	32 (22–65)

	*N* (%)	*N* (%)	*N* (%)
*Sexual orientation in affirmed gender*			
Heterosexual	53 (47.3%)	26 (32.1%)	1 (14.3%)
Homosexual	19 (17.0%)	18 (22.2%)	0 (0.0%)
Bisexual	27 (24.1%)	26 (32.1%)	2 (28.6%)
Other	13 (11.6%)	11 (13.6%)	4 (57.1%)
*Education*			
None	4 (3.5%)	3 (3.3%)	0 (0.0%)
Compulsory school	17 (15.0%)	12 (13.3%)	0 (0.0%)
Apprenticeship	38 (33.6%)	27 (30.0%)	4 (57.1%)
High school diploma	31 (27.4%)	23 (25.6%)	1 (14.3%)
University	11 (9.7%)	17 (18.5%)	2 (28.6%)
Other	12 (10.6%)	8 (8.9%)	0 (0.0%)
*Nationality*			
Austria	84 (77.1%)	72 (85.7%)	3 (60.0%)
Germany	10 (9.2%)	6 (7.1%)	0 (0.0%)
Italy	3 (2.8%)	3 (3.6%)	1 (20.0%)
Switzerland	1 (0.9%)	0 (0.0%)	0 (0.0%)
Luxembourg	2 (1.8%)	0 (0.0%)	1 (20.0%)
Other	9 (8.3%)	3 (3.6%)	0 (0.0%)

### Age at Gender Identity Development Milestones

**Fig. 1:**
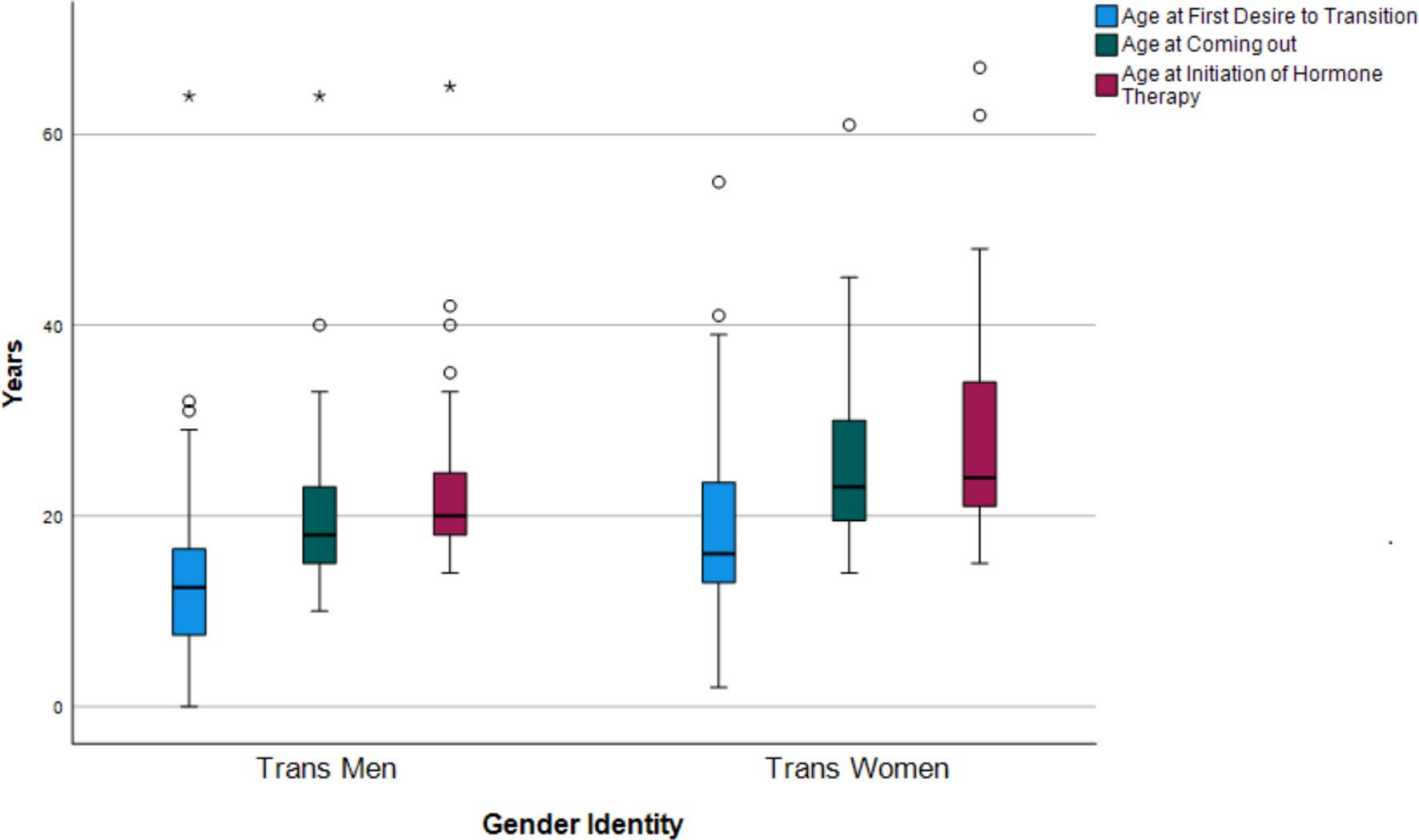
Age at milestones stratified by gender identity

Table 2 and Fig. 1 show the ages (means and SDs) at the three GID milestones by gender identity. At each milestone trans men were younger compared to trans women (all *p* < 0.001).

**Table 2:** Age at gender identity milestones

	Trans men	Trans women	Gender diverse
Age at first desire to transition	13.9 (9.0)	18.8 (10.1)	17.4 (9.4)
	*N* = 98	*N* = 70	*N* = 5
Age at coming out	20.0 (7.2)	25.3 (9.1)	23.5 (7.6)
	*N* = 100	*N* = 70	*N* = 4
Age at initiation of hormone therapy	22.6 (7.4)	28.6 (10.1)	32.8 (17.4)
	*N* = 97	*N* = 76	*N* = 5

Means and standard deviations for age at the three gender identity milestones, separated by gender identity

**Fig. 2:**
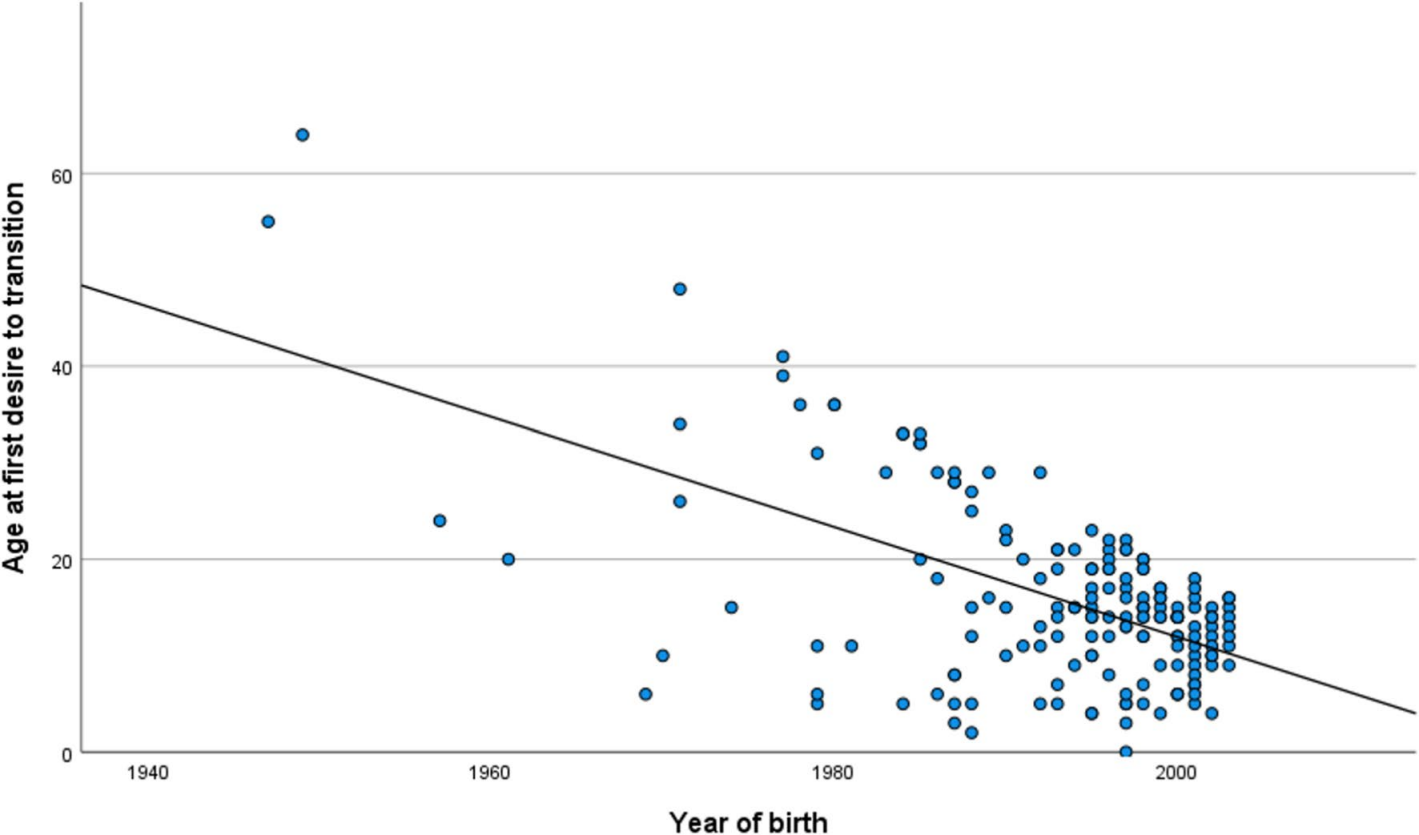
Association between year of birth and age at first desire to transition

Year of birth significantly predicted age at first desire to transition in a linear regression model (*B* = − 0.57, 95% CI (− 0.69 to − 0.45), *p* < 0.001; see Fig. 2).

**Fig. 3:**
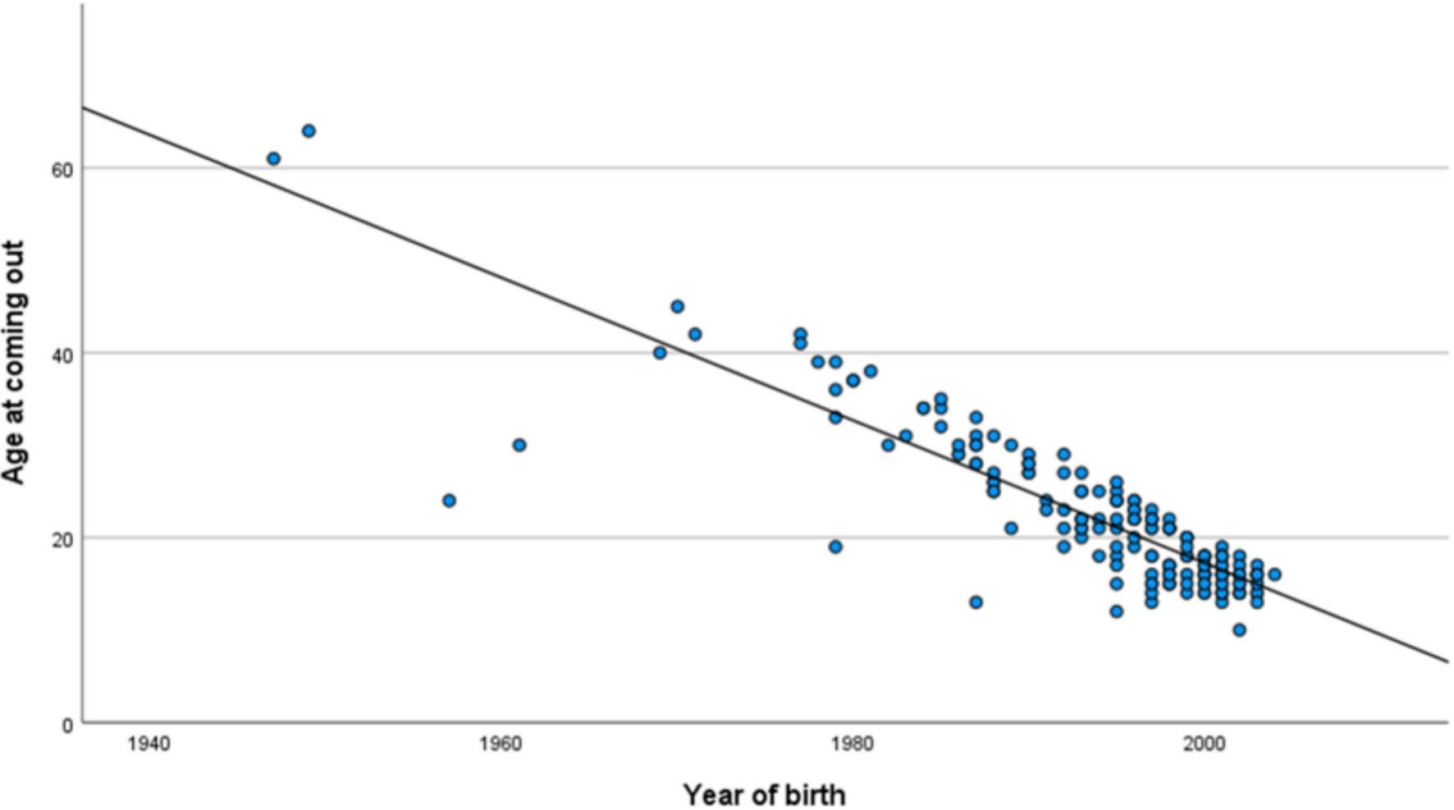
Association between year of birth and age at coming out

Similarly, a significant association between year of birth and age at coming out was seen (*B* = − 0.77, 95% CI (− 0.84 to − 0.71), *p* < 0.001; see Fig. 3).

**Fig. 4:**
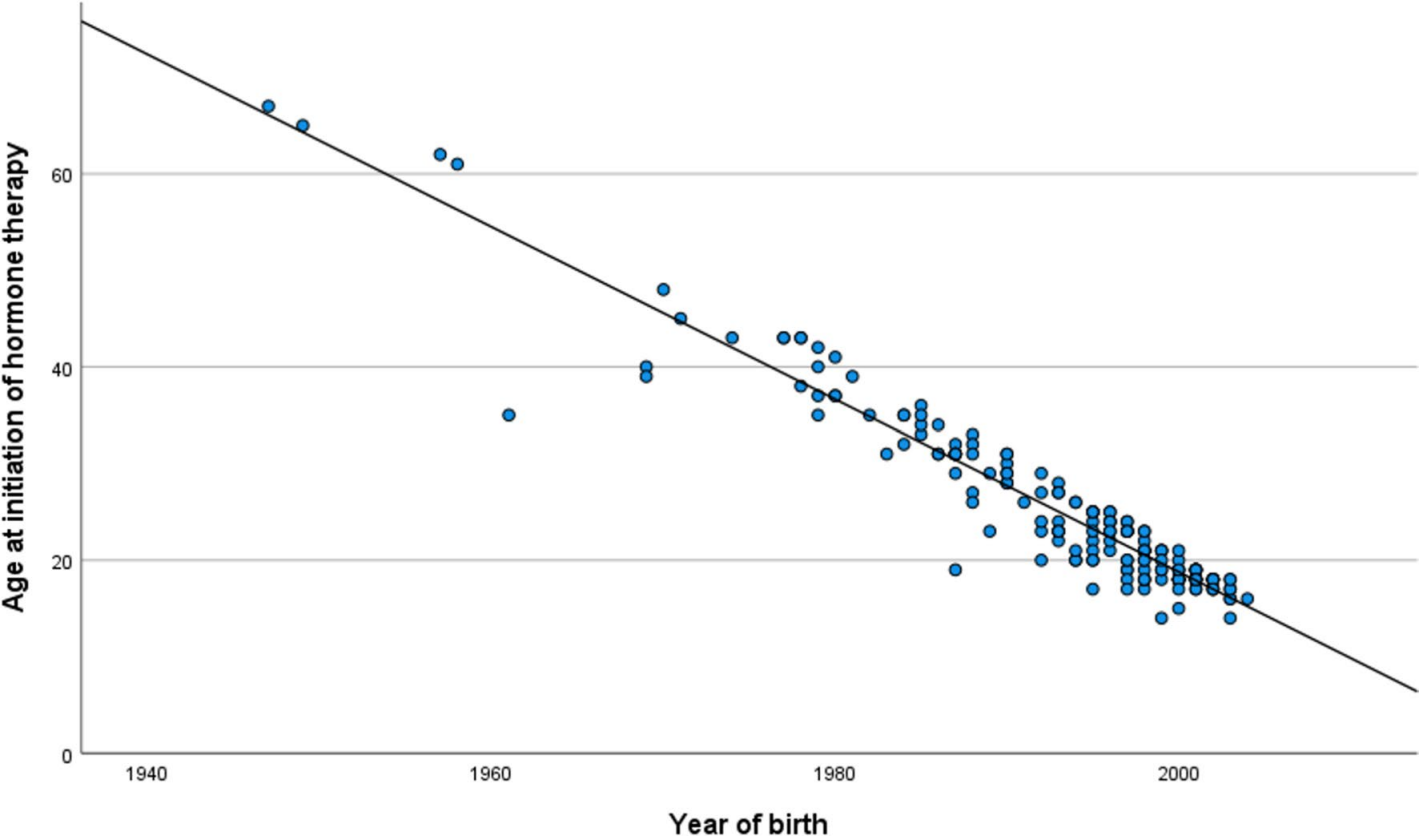
Association between year of birth and age at initiation of hormone therapy

The age at which individuals initiated hormone therapy was also modeled using linear regression with year of birth as the predictor with a statistically significant regression coefficient of − 0.89 (95% CI (− 0.93 to − 0.85), *p* < 0.001; see Fig. 4). Later birth years were associated with earlier ages at first desire to transition, coming out, and the initiation of therapy. A summary of the three linear regression models is presented in Table 3.

**Table 3:** Linear regression analyses of the associations between year of birth on age at gender identity milestones

	*B* (95% CI)	*p*
Age at first desire to transition	− 0.57 (− 0.69 to − 0.45)	< 0.001
Age at coming out	− 0.77 (− 0.84 to − 0.71)	< 0.001
Age at initiation of hormone therapy	− 0.89 (− 0.93 to − 0.85)	< 0.001

Univariable linear regression models examining the associations of year of birth (independent variable) and the age at which individuals experience gender identity development milestones (dependent variable). For each model, the regression coefficient (*B*) with 95% confidence interval (95% CI) and the *p*-value (*p*) are displayed

To further investigate the temporal dynamics of milestone progression, linear regression models were conducted on the time spans between them (Table 4). These analyses have shown significant associations between year of birth and the time spans between (1) first desire to transition and coming out (*B* = − 0.22, 95% CI (− 0.33 to − 0.11), *p* < 0.001), (2) coming out and initiation of hormone therapy (*B* = − 0.10, 95% CI (− 0.16 to − 0.04), *p* < 0.001) and (3) first desire to transition and initiation of hormone therapy (*B* = − 0.33, 95% CI (− 0.45 to − 0.21), *p* < 0.001), respectively.

**Table 4:** Linear regression analyses of the associations between year of birth on the time spans between gender identity milestones

	Mean (SD), years	*B* (95% CI)	*p*
First desire to transition—coming out	6.5 (7.1)	− 0.22 (− 0.33 to − 0.11)	< 0.001
Coming out—initiation of hormone therapy	2.3 (3.9)	− 0.10 (− 0.16 to − 0.04)	< 0.001
First desire to transition—initiation of hormone therapy	8.9 (8.0)	− 0.33 (− 0.45 to − 0.21)	< 0.001

Mean and standard deviation (SD) for time spans between gender identity development milestones and univariable linear regression models examining the associations of year of birth (independent variable) and time spans between gender identity development milestones (dependent variable). For each model, the regression coefficient (*B*) with 95% confidence interval (95% CI) and the *p*-value (*p*) are displayed

### Gender Identity

Additionally, logistic regression analysis revealed a relationship between birth year and gender identity (*n* = 90 trans women vs. *n* = 122 trans men and gender diverse individuals). Participants who were born earlier had significantly higher odds of identifying as trans women compared to those born in more recent years (OR = 0.95, 95% CI (0.92–0.98), *p* < 0.001).

**Fig. 5:**
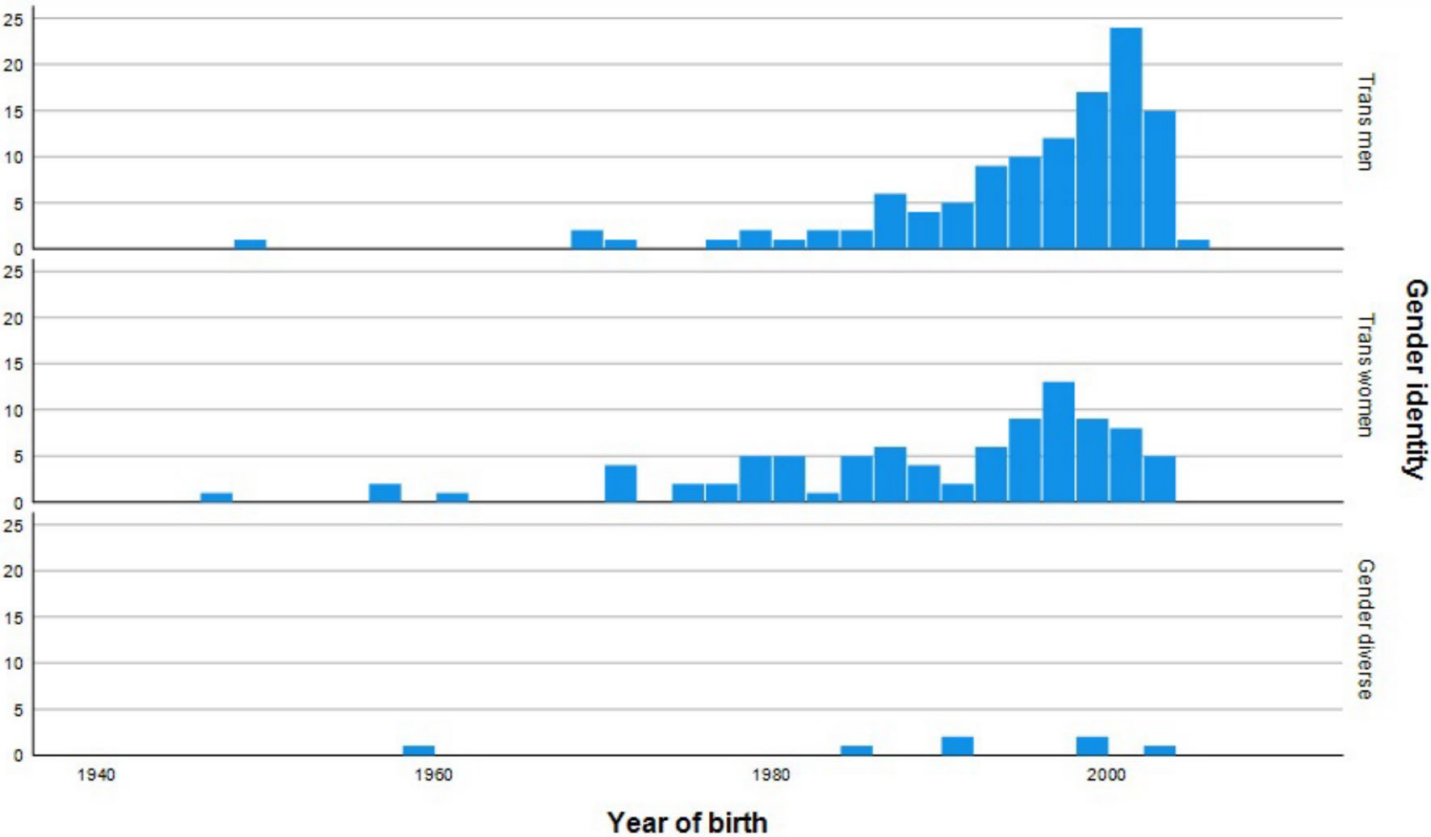
Distribution of birth years among participants seeking gender-affirming hormone therapy by gender identity

Figure 5 illustrates the distribution of birth years among participants seeking gender-affirming hormone therapy, stratified by gender identity (trans men, trans women, gender diverse). The histograms revealed a general increase in individuals seeking therapy over time, with a notable surge in trans men born in the late 1990s to early 2000s, resulting in a right-skewed distribution indicating a younger age profile for this group. Trans women also showed an increasing trend in frequency, but their birth year distribution is more dispersed. The number of gender diverse participants in our sample was comparatively low. Overall, the data indicated a trend toward younger individuals seeking hormone therapy across all gender identities, most prominently in trans men.

## Discussion

This study provided insight into the impact of age on the process of coming out as a TGD person. A significant negative relationship between year of birth and the age at which TGD individuals reached the examined milestones was demonstrated. Later birth years were associated with earlier ages at first desire to transition, coming out, and the initiation of therapy. Each subsequent year of birth was associated with a decrease of 0.57 years (6.84 months) for first desire to transition, 0.77 years (9.24 months) for coming out, and 0.89 years (10.68 months) for initiation of hormone therapy.

The strength of these associations varied with the strongest association observed for the initiation of hormone therapy. Analysis by gender identity further indicated an increasing trend in individuals seeking hormone therapy in younger cohorts and with younger participants more commonly identifying as trans men, whereas each additional birth year decreased the likelihood of identification as a trans woman by 5%. Trans men reached all examined milestones at significantly younger ages than trans women, as confirmed by both *t*-tests and descriptive comparisons of means and standard deviations. Furthermore, an association was demonstrated between year of birth and time spans between gender identity milestones, showing a shortening in time intervals in younger participants. These findings suggested a temporal shift toward earlier and more accelerated affirmation of gender identity. This pattern of faster milestone attainment was evident in our data, which showed a significant negative correlation between year of birth and the age at which each milestone is accomplished. The average time interval between the first desire to transition and coming out was 6.5 years (SD 7.1), and only 2.3 years (SD 3.9) between coming out and starting hormone therapy. Regression analysis further highlighted that each later birth year was associated with a significant reduction in these intervals (*B* coefficients all negative, *p* < 0.001).

Such temporal compression may be consisted with shifting sociocultural conditions, including greater visibility of TGD identities, more accessible information, a growing network of peer and community supports, and evolving standards of affirmative care and medical pathways, which could plausibly contribute to shorter intervals between coming out and initiation of hormone therapy. For example, until 2009, people in Austria were required to undergo genital surgery in order to legally change their name and gender markers on certificates and legal documents and until 2006, individuals had to be divorced before they could legally transition. Financial factors may also have impacted the time between reaching milestones; even in the late 1990s, insurance companies did not cover any transition-related costs (e.g., assessments, psychotherapy, hormone therapy, and surgeries). Significant changes in legal regulations in recent decades may have had a considerable impact on the time spans between milestones. For instance, for many years, only one institution in Vienna was authorized to conduct the necessary evaluations prior to gender-affirming care. Subsequently, the number of institutes issuing these assessments increased slowly.

Our findings of both accelerated milestone attainment and compressed intervals between the first desire to transition, coming out, and initiation of hormone therapy in younger cohorts are echoed in recent research on sexual minority development. Bishop et al. (2024) offered strong evidence that generational cohort effects play a major role in the timing of sexual identity milestones: in harmonized samples across three sociohistorical periods, more recent cohorts of sexual minority adolescents reported much earlier ages for key milestones—particularly self-identification and disclosure—compared to earlier cohorts. Importantly, the study separated maturational age influences from genuine cohort effects by using age-matched samples, confirming that sociohistorical context drives earlier disclosure and identity affirmation. Similarly, the systematic review and meta-analysis by Hall et al. (2021) highlighted substantial heterogeneity in the age and sequence of sexual orientation identity milestones but also demonstrated a general trend toward earlier and closer milestone timing for younger generations. The meta-analysis found that coming out typically occurs in late adolescence or young adulthood—with generation and birth cohort exerting a significant effect. As Bishop et al. (2024) demonstrated, maturational context matters, but even when controlling for age, cohort effects on milestone timing remain robust. Our own data, showing the negative regression coefficients for birth year and milestone intervals, fit precisely into this picture: shorter intervals and earlier milestone ages reflected not only improvements in support and visibility, but also the ongoing adaptation of identity development patterns to shifting sociohistorical landscapes.

Our data confirmed previous studies conducted in an Italian cohort of *n* = 197 TGD individuals split into Generation Z (born 1997–2012), Millennials (born 1981–1996) and Generation X (born 1965–1980). First insights into TGD identity, self-identification as TGD and coming out were assessed. Interestingly, no age-related differences were found for first insights, but Generation Z showed earlier coming out than Generation X and Millennials. Baby Boomers (born 1946–1964) were not included (Scandurra et al., 2021). Contrary to the approach in this study, the authors utilized generation as foundation for their calculations. The classification of subjects according to generational categories has been shown to attenuate the impact of age in comparison with the calculation based on year of birth. Consequently, studies employing generation-based calculation methods may have yielded misleading results (Costanza et al., 2023).

In another generation-based cohort of *n* = 3796 TGD individuals aged 25–98 (Millennials, Generation X, Baby Boomers and older), Wilkinson et al. (2018) reported on first recognition of gender difference, first identification as transgender, and first living as transgender in relation to educational attainment by using data from the National Discrimination Survey (2008–2009). In contrast to our study, there were no age differences in first recognition. However, younger generations also showed earlier first identification and living as a TGD. Because the data were collected in 2008, Generation Z was not included, nor was the time interval between GID milestones. Puckett et al. (2022) also explored the relationship between age at achievement of milestones and generational cohorts. Their findings indicated that older generations were more likely to self-identify as trans women and commence gender affirmation procedures and medical care at a later stage in life.

A further focus of our research was the relationship between gender identity and birth year. Participants born earlier exhibited significantly higher odds of identifying as trans women, whereas younger participants were more likely to identify as trans men. These results aligned with findings by Puckett et al. (2022) and Scandurra et al. (2021), who likewise reported a shift toward a higher proportion of trans men over time. It was also consistent with recent epidemiological research, which demonstrated an overall increase in both identification as TGD and specifically as trans man (Fisher et al., 2024; Jarvis et al., 2025; Lagos, 2022; Nolan et al., 2019). Leinung and Joseph (2020) not only stated these demographic changes but also that the age at start of GAHT was significantly higher for trans women.

There are a number of factors that need to be considered when explaining these age-related differences. For older trans women, paths to visibility and transition were often shaped by binary and medicalized frameworks that primarily recognized and organized care around trans female identities, reflecting a historical context in which the concept and visibility of trans men were far less established. Coming out was frequently delayed until after important life milestones such as starting a career or raising children (Fabbre, 2015). Many navigated their gender identity in an environment characterized by significant stigma and limited access to affirming resources. Recent qualitative research highlighted how successful aging for transgender individuals often hinges on resilience, adaptive coping, and the pursuit of authenticity later in life (Sloan & Benson, 2022). Younger cohorts have grown up in a more inclusive environment. Greater visibility of trans and gender diverse identities, development of peer support networks and easier access to information and health care have enabled earlier and more confident self-identification among trans men. These sociocultural changes have coincided with improvements in healthcare access and ethical frameworks that emphasize autonomy and self-definition. Such frameworks, often reflected in the principles of informed consent and affirmative care models, aim to empower individuals in their gender-affirming journeys. The observed age-related pattern therefore reflected not only demographic variation but also the evolving ethical landscape that has reshaped what it means to seek and live as one’s affirmed gender.

In our cohort, the overall frequency of gender diverse individuals was low (3.3%). While community-based online surveys have reported rates of non-binary gender identities ranging from 35 to 40% (James et al., 2016; Reisner & Hughto, 2019), clinic-based studies typically showed substantially lower percentages. A recent study from Germany yielded comparable results, with non-binary gender identities accounting for approximately 10% of the participants at a single center specialized in counseling and treatment for children and adolescents. A non-binary gender identity has been shown to be significantly associated with a lower likelihood of seeking gender-affirming medical treatment or with a preference for puberty-blockers only (Herrmann et al., 2024). As in the present study, data collection occurred in the same clinical context where participants received psychological or medical support. This might have led to withholding or underreporting a non-binary gender identity out of fear of jeopardizing access to care. Furthermore, gender diverse individuals were more likely to experience higher rates of discrimination compared to those identifying within the binary spectrum (Taube & Mussap, 2024). Such anticipation of discrimination and concerns about being denied GAHT could therefore contribute to the relatively low proportion of gender diverse participants observed.

The educational attainment of the study population differed from that of the general Austrian population as reported by Statistik Austria (2022). While 13.8% of the participants reported compulsory schooling as the highest educational attainment (in Austria compulsory schooling ends at the end of the 8th grade), 17.1% of the Austrian population did so, including those without completed compulsory education. In line with national data, the proportion of early school leavers aged 18–24 who discontinued education or training was 8.6% in 2023. The low rate of 3.3% persons without compulsory schooling in the current study therefore appears consistent with national trends. However, the study population was underrepresented in those holding a high school diploma or university degree (4-year degree) compared to the general Austrian population. Caution is warranted when interpreting these findings, as Statistik Austria calculates educational attainment for individuals aged 25–64 years, whereas the study included participants aged 20–76. Thus, younger individuals may still be in the process of completing higher education. Nevertheless, structural and psychosocial factors may also have contributed to lower educational achievement among transgender individuals. Previous research has shown that TGD individuals experience elevated rates of adverse childhood experiences, discrimination, and mental health problems such as depression and anxiety, all of which can interfere with educational continuity and attainment (Di Fini et al., 2025; Feil et al., 2023).Wilkinson et al. (2018) further demonstrated that those who experienced transgender identity milestones during adolescence were less likely to complete a 4-year degree. This emphasizes the educational barriers associated with early stigma and minority stress during a critical developmental period, as well as the impact of mental health impairments on educational attainment.

The study had some limitations: Since only individuals who had consulted a center specializing in the medical treatment of TGD individuals and therefore wanted to start or had already started GAHT were considered, individuals without a desire for treatment were automatically excluded from the study. This naturally influenced the representativeness of the study. In considering this matter, it is important to note that the recruitment process at a single center represents another potential limitation. The study design was based on retrospective self-reporting, which may have introduced potential recall and social desirability biases. Moreover, each milestone was assessed using a single item in the questionnaire, thus failing to provide a more comprehensive analysis of the individual coming out trajectories. The order of milestones in our analysis is based on binary transition models. However, there is increasing evidence that TGD individuals navigate varied and occasionally nonlinear trajectories that may not align with distinct sequential stages. Gender diverse individuals often experience identity formation, disclosure, and medical processes differently from how they are presented in conventional models. We recognize that the model does suggest a normative developmental trajectory and that it may inadequately represent the lived experiences and developmental intricacies of the full spectrum of TGD identities. Finally, we want to acknowledge that birth year and age at milestone are generally mathematically related, which might have overestimated the strength of the observed association.

Missing data in GID milestones were observed but did not exceed 18.5% (see Appendix A). It remained unclear whether the missing data were due to not having reached the specific milestone, not remembering when the milestone was reached, or respondents choosing not to answer. Since the questionnaire did not include an option to provide free text responses regarding milestones, no conclusions could be drawn.

## Conclusion

This study highlighted the need for age-sensitive approaches in supporting TGD individuals. By recognizing and addressing the unique challenges faced by different age groups, we can foster more inclusive and supportive environments that promote their well-being and acceptance in society. Future research should continue to explore generational dynamics and develop tailored interventions and treatment strategies.

## Data Availability

Data and material are available from the authors at request.

## Appendix A: Response and Missing Data Rates

**Table:**

	Response rate *n* (%)	Missing data rate (%)
Year of birth	212 (100%)	0 (0%)
Gender identity	212 (100%)	0 (0%)
Sexual orientation	200 (94.3%)	12 (5.7%)
Education	210 (99.1%)	2 (0.9%)
Nationality	198 (93.4%)	14 (6.6%)
First desire to transition	173 (81.6%)	39 (18.4%)
Coming out	174 (82.1%)	38 (17.9%)
Initiation of hormone therapy	178 (84.0%)	34 (16.0%)
